# Ruling in the diagnosis of methanol intoxication in a young heavy drinker: a case report

**Published:** 2012-09-25

**Authors:** D Anyfantakis, EK Symvoulakis, EV Cristodoulakis, G Frantzeskakis

**Affiliations:** * Primary Health Care Centre of Kissamos, Chania, Crete, Greece; ** University Hospital of Heraklion, Crete, Greece; *** Department of Ophthalmology, General Hospital of Rethymnon, Crete, Greece; **** Primary Health Care Centre of Spili, Rethymnon, Crete, Greece

**Keywords:** methanol intoxication, visual loss, diagnosis, management

## Abstract

Methanol poisoning is a relatively rare but potentially serious medical emergency. Toxicity results when methanol is successively oxidized to the active metabolites formaldehyde and formic acid. We report a case of a 23-year-old male, a high daily alcohol consumer, who attended the local primary health care centre complaining of sudden visual loss. A presumed diagnosis of methanol intoxication was suggested based on the patient’s visual impairment and the history of alcohol ingestion. Specific therapy was initiated before a definitive diagnosis. Gas chromatographic determination of methanol levels confirmed the initial diagnostic suspicion. In this case, prompt recognition of methanol intoxication and treatment conditioned a favorable clinical outcome. Given that timely diagnosis and antidote administration are crucial issues in terms of prognosis, we underline the necessity for physicians to be alert for entities provoked by rare environmental factors.

## Introduction

Methanol or wood alcohol is a colorless liquid with a slightly sweeter odor than ethanol [**1**]. It is commonly used in a variety of industrial solvents, such as antifreeze, paint remover and in the preparation of adulterated beverages [**1**] leading to ‘endemics’ of poisoning [**2**]. Methanol intoxication represents a rare clinical presentation [**1**] although the total number of cases is probably underreported [**3**]. It constitutes a severe form of poisoning [**1**] that, if left untreated, is associated with significant mortality and visual deficits among survivors [**3**]. Ingestion of only 15 ml of 40% methanol may be fatal [**2**]. We report a case of visual impairment from methanol poisoning in a 24-year-old Greek male.

## Case presentation

 A 24-year-old male, a permanent resident from a rural area in Crete, was admitted to a rural primary health care centre with blurred vision and epigastric pain. He was anxious and hyperpneic. During the last days, the patient reported a repetitive abuse of local home-distilled alcoholic drinks not being able to offer specific information on the exact conditions of alcohol consumption (place and quantity).

 Vital signs were as it follows: blood pressure, 150/90 mmHg; pulse, 115 beats/min; respiration, 28 breaths/min; temperature, 35,8oC. There was no odor of ethanol in his breath. Inspection did not reveal any traumatic lesion. The remainder of the physical examination was unremarkable. Electrocardiogram showed a sinus rhythm. Visual deficit in combination with alcohol ingestion raised the suspicion of methanol poisoning. After consultation with the poison information center of Greece a transfer to a secondary care centre was immediately arranged. 

 On admission, the ophthalmologic examination revealed an almost total decline in visual acuity and central scotoma in both eyes. His pupils were normal with a moderate reaction to light. Ocular fundus was bilaterally normal. Since toxicological assays for toxic alcohols were not available in the hospital, blood samples were obtained and transferred to a specialized clinical centre. Thirty minutes after the arrival, the initial laboratory evaluation revealed a complete normal blood count. Serum chemistries disclosed serum sodium of 133 mEq/L, creatinine of 101.6 μmol/l, potassium of 5 mEq/L, chloride of 101 mEq/L, urea of 32mg/dl and glucose of 7.3mmol/l. Liver functions tests were normal. The patient’s arterial blood gas results when he was breathing ambient air were: pH 7.25; PO2 of 118mmHg, PCO2 of 16 mmHg and bicarbonate level of 9 mmol/L. Serum anion gap calculated by the formula: [sodium (Na+)-[chloride (Cl-)+bicarbonate (HCO3-)][**4**] was 23 mEq/l (normal range: 8-16mEq/L) [**4**]. The measured serum osmolality (by freezing point depression) was of 316 mOsm/Kg. The calculated serum osmolarity by the equation 2[Na+ (meq/L)]+[BUN(mg/dL)]/2,8+ [Glucose (mg/dL)]/18 [**4**] was of 278,66 mOsm/Kg, yielding an osmolar gap of 38,66 mOsm/Kg. Magnetic resonance imaging and computed tomography scan of the brain did not reveal any abnormalities.

 Antidotal therapy was initiated within 1h after hospital admission. A loading dose of 600mg/kg (1,8mL/kg) ethanol 43% (as fomepizole was not routinely included in the hospital formulary) was administrated via a nasogastric tube followed by a continuous infusion of 154mg/kg/h (0,46ml/kg/h). Taking into account the visual impairment, the patient was transferred to the intensive care unit for the institution of hemodialysis. A four-hour course of hemodialysis was instituted. During this interval, the dosing schedule of ethanol infusion was adjusted to 257mg/kg/h (0,77ml/kg/h). Since the patient became lethargic during therapy, ethanol was discontinued and substituted by fomepizole, which was already obtained. A loading dose of 15mg/kg fomepizole was administered intravenously (over a 30-minutes period) 6 hours after hospital admission, followed by 10mg/kg every 12 hours. An intravenous dose of 50mg of folinic acid was also given every 6 hours. The diagnosis of methanol intoxication was confirmed when toxicological screening with gas chromatography disclosed serum methanol concentration of 14.9 mmol/L (48mg/dl) on hospital admission. Serum ethanol levels were not detected. During therapy, ophthalmologic examinations were performed daily. The patient presented a progressive resolution of visual symptoms and a complete recovery of his visual acuity; on the fourth day after initiation of therapy he had best corrected visual acuity 0.15/0.3 (Snellen) in the right and left eye respectively, while on the sixth day he had 0.8 in both eyes. Serum chemistry and arterial blood gases on the second and third day of hospitalization were within normal ranges. The patient was discharged after 4 days of hospital stay. Laboratory screening for serum methanol on discharge was negative. A follow up ophthalmologic evaluation within one month revealed a normal visual acuity (1.0 in both eyes) and recovery of the visual field from scotoma.

## Discussion

Methanol intoxication occurs through unintentional or suicidal ingestion as well as after attempted inebriation [**3**]. Alcoholics and prisoners are considered high-risk groups that often substitute ethanol with methanol-contained substances [**5,6**]. Pure methanol is not toxic, until it is metabolized by alcohol dehydrogenase to formaldehyde and subsequently to formic acid [**6**]. Accumulation of the later has been reported to be the main cause of the adverse effects typically observed on methanol intoxication such as metabolic acidosis, neurological impairment and ocular injury [**6**]. Remarkably, the formic acid’s serum concentration level is considered a poor prognostic parameter correlated with high toxicity [**7**].

Toxical symptoms usually occur after an asymptomatic latent period of 8 to 24 hours following methanol ingestion [**1**]. In case of concomitant ethanol ingestion, the onset is usually delayed by 24 hours [**6**]. Clinical manifestations of methanol poisoning are non-specific [**5**] making the diagnosis challenging [**8**]. Early signs include abdominal discomfort, nausea, vomiting, and a mild central nervous system depression [**1**]. Late onset signs include anion gap acidosis, neurological dysfunction and ophthalmological disturbances [**5**]. Visual symptoms are the most specific clinical features [**5,8**] and range from photophobia and a decline of visual acuity to complete blindness [**1**].

Anion and osmolar gap (the difference between measured serum osmolality and calculated serum osmolarity) are used routinely in the evaluation of patients with a suspicion of toxic alcohol poisoning [**5**]. Laboratory evidence of metabolic acidosis along with the elevation of anion and osmolar gap are features that suggest a diagnosis of methanol intoxication [**8**]. However, an isolated normal value of anion or osmolar gap is of limited diagnostic accuracy [**6**]. This happens because these markers follow a chronological variation. Early after methanol ingestion, a maximum elevation of the osmolar gap usually occurs. Later in the course of poisoning, as metabolic degradation of methanol proceeds, osmolar gap decreases and may be normal while anion gap increases [**6**].

Definitive diagnosis requires measurement of the serum concentration of methanol by the gold standard test which is gas chromatography and confirmation of an elevated methanol level (> 6mmol/L or 20mg/dL) [**6**]. The appropriate management relies on the prompt inhibition of enzymatic oxidation of methanol to formic acid through antidote provision (fomepizole or ethanol) [**3**], which also represents an important prognostic factor associated with recovery from visual impairment [**9**]. Noteworthy, even though ocular complications are the most specific clinical signs in methanol poisoning, they are underestimated and progress over time even after hospital discharge [**10**]. Regarding the therapy, although there is a lack of clinical evidence to confirm the superiority of fomepizole over ethanol, the former has been reported to be the antidote of choice in methanol poisoning [**6**]. Adjunctive hemodialysis and intravenous administration of folinic acid may be useful in order to enhance the elimination and increase the metabolism of formic acid respectively [**6**].

In this case, the hospital where the patient was admitted was not equipped to perform toxicological assays and a definitive diagnosis would require a waiting for confirmation of blood results. However, the decision to initiate a specific treatment was made only based on the patient’s high daily alcohol consumption history, ocular symptoms and anion and osmolar gap elevation. This case highlights the necessity for physicians to always cultivate a ‘detective judgment’, especially for rare conditions and when patients’ clinical presentation cannot support a likely diagnosis.

## References

[1] Williams  GF, Hatch  FJ, Bradley  MC (1997). Methanol poisoning: a review and case study of four patients from central Australia. Aust Crit Care.

[2] Bennett  IL Jr, Cary  FH, Mitchell  GL (1953). Acute methyl alcohol poisoning: a review based on experiences in an outbreak of 323 cases.. Medicine (Baltimore).

[3] Brent  J (2009). Fomepizole for ethylene glycol and methanol poisoning. N Engl J Med.

[4] Boyle  JS, Bechtel  LK, Holstege  CP (2009). Management of the critically poisoned patient. Scand J Trauma Resusc Emerg Med.

[5] Kruse  JA (1992). Methanol poisoning. Intensive Care Med.

[6] Barceloux  DG, Bond  GR, Krenzelok  EP (2002). American Academy of Clinical Toxicology Ad Hoc Committee on the Treatment Guidelines for Methanol Poisoning. American Academy of Clinical Toxicology practice guidelines on the treatment of methanol poisoning. J Toxicol Clin Toxicol.

[7] Brent  J, McMartin  K, Phillips  S (2001). Methylpyrazole for Toxic Alcohols Study Group. Fomepizole for the treatment of methanol poisoning. N Engl J Med.

[8] Becker  CE (1983). Methanol poisoning. . J Emerg Med.

[9] Sivilotti  ML, Burns  MJ, Aaron  CK (2001). Reversal of severe methanol-induced visual impairment: no evidence of retinal toxicity due to fomepizole. J Toxicol Clin Toxicol.

[10] Paasma  R, Hovda  KE, Jacobsen  D (2009). Methanol poisoning and long term sequelae-a six years follow-up after a large methanol outbreak. BMC Clin Pharmacol.

